# Aortic valve surgery: how reliable are health information websites?

**DOI:** 10.3399/bjgpopen17X100665

**Published:** 2017-03-15

**Authors:** Ming Yi Lai, Hilary McDermott, John B Chambers

**Affiliations:** 1 Medical Student, University of Cambridge, Cambridge, UK; 2 Honorary research fellow, Guy's and St Thomas' Hospital NHS Foundation Trust, London, UK; 3 Patient, Guy’s and St Thomas’ Hospital NHS Foundation Trust, London, UK; 4 Professor of Clinical Cardiology, Guy’s and St Thomas’ Hospital NHS Foundation Trust, London, UK

**Keywords:** aortic valve replacement, cardiology, internet information

## Abstract

**Background:**

Aortic valve replacement is one of the most common cardiac operations currently performed. Patients increasingly use the internet for information about their diagnosis and it would therefore be important to know how reliable this is.

**Aim:**

To determine the reliability of internet information on aortic valve replacement surgery.

**Design & setting:**

This was a web-based project scoring sites that might be accessed by a patient.

**Method:**

The first 50 websites found on each of the four most popular search engines in the UK were viewed, as well as the first 50 videos found on the most popular video-host website. Eligible websites were assessed according to seven positive criteria and three negative criteria, giving a possible range of scores from −6 to 14.

**Results:**

There were 79 sites and the median score was 5 (range −1 to 14). There were statistically significant differences between organisation/educational sites with score 7 (2 to 14), hospital sites with score 2 (−1 to 10), commercial sites with score 2.5 (0 to 9) and videos with score 5 (2 to 11). The highest scores went to three NHS sites (score 13 or 14), .gov sites (median score 8.5) and Health On the Net Foundation (HON) accredited sites (median score 7).

**Conclusion:**

Information on the internet about aortic valve replacement is variable but NHS sites provide the most reliable information.

## How this fits in

Aortic valve replacement is an increasingly common procedure and results are improved if patients are involved in decision making if they wish to be. Patient empowerment requires information for which the internet is a major source. However, the reliability of this information is variable and clinicians need to direct patients to the best sites since these may not appear on a brief internet search. Supplementary information will also need to be given since most websites are limited in scope.

## Introduction

Aortic valve replacement surgery is increasingly common as our population ages. In 2008, there were 7280 aortic valve replacement operations in the UK and Ireland compared with 22 808 isolated coronary bypass procedures.^^1^ Not all patients wish to be involved in discussion of the timing of surgery, or the choice of valve type.^2^ However, if they do, an informed discussion may improve quality of life after surgery.^2,3^ An estimated 68% of British patients will search for medical information online4^ may then request a discussion with their GP or practice nurse. The quality and reliability of general information available on the internet is known to vary greatly^4–7^ but there are few critical appraisals of medical sites. The research team assessed the information that might be accessed on the internet by a patient needing an aortic valve replacement.

## Method

The authors used the four most popular search engines in the UK (Google, Yahoo, Bing, and Ask), which cover >98% of search engine usage in the UK.^8^ In addition, a video search on YouTube was performed. The phrase 'aortic valve replacement' was searched. To prevent bias in the search algorithm, an incognito window was used to prevent cookies and search history from influencing search results.

The first 50 links from each search engine and YouTube were followed. Of the 250 results, there was significant overlap (especially between Google, Yahoo, and Bing searches), leaving 165 unique websites. Of these, 86 sites were irrelevant, such as advertisements, forums, other recommended searches, and sites detailing other procedures (for example, aortic root replacement). There were 79 relevant websites remaining. These were categorised as:

- organisational/educational (for example, published by a large charitable organisation, university or other information provider);
- hospital-based;
- commercial (for example, published by a prosthetic valve manufacturer); and 
- video.

The sites were also categorised according to top level domains (TLD): .com, .co.uk, .org, .edu, .gov and variations on these, on the assumption that patients c use these as a guide to quality.

There were no previously published scoring systems available so the researchers designed one (Appendix 1) based on:

- the types of information known to be important and likely to be of interest to a patient;
- a previously published test of reliability;^9^ and 
- adaptations of HON^10^ guidelines, relevant to the topic studied.

Each website was assessed according to 10 criteria and given scores from 0 to 2 for each positive attribute and 0 to −2 for each negative attribute assessing accuracy, reliability, and appropriateness of information (Appendix 1). The minimum achievable score was −6 and the highest was 14; the higher the score the better the site. Bias was defined as recommending a procedure or type of prosthetic valve outside established guidelines.^11,12^ There were three patient forums associated with these websites. These frequently contained inaccurate information or unhelpful subjective comments, for example 'a metal valve can start to fail after 25 years' and 'I didn’t want a pig to die so I could have its valve, only to have it taken out some years on; it seemed wasteful and wrong.' These were not assessed within this study as these forums do not vet their responders, and the study was confined to sites that a patient might assume are authoritative and comprehensive.

Websites were checked for their accreditation by HON by keeping the 'HON toolbar' open.^12^ HON is a non-profit, non-governmental organisation, accredited by the Economic and Social Council of the United Nations created in 1995 which promotes and guides the deployment of useful and reliable online health information, and its appropriate and efficient use. Although HON does not assess medical accuracy, validity, or appropriateness, it is a good guide to the quality and accountability of websites according to eight standards: information must be authoritative; the purpose of the website must be known; confidentiality must be respected; information must be documented (that is, referenced and dated); claims must be justified (for example, with scientific evidence); contact details must be made available; funding sources must be disclosed; and, there must be an advertising and editorial policy available for viewers to see.

## Statistical analysis

The scores were not normally distributed using the Kolmogorov-Smirnov normality test (*P*<0.05) so medians and ranges were reported. Comparisons between scores in the categories were made using the Mann-Whitney U-test and Kruskal-Wallis one-way analysis of variance.^13^

## Results

**Figure 1.**
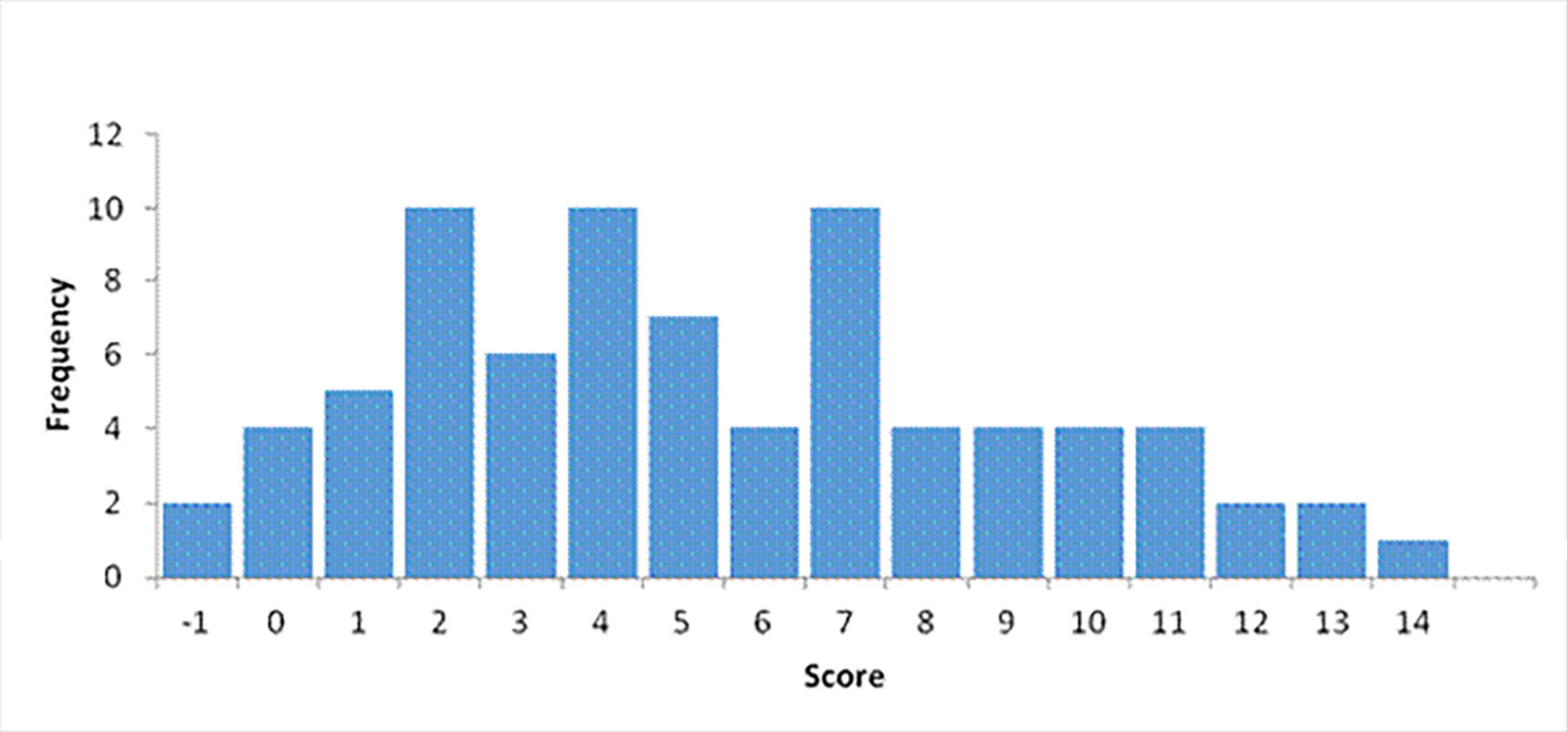
Histogram showing distribution of scores.

Of the 79 sites assessed (Appendix 2), 33 (43%) were organisational or educational, 24 (30%) hospital-based, 8 (10%) commercial, and 14 (17%) videos. The median score was 5 (range −1 to 14) as seen in Figure 1. Different search engines produced different results with ask.com providing 67% of the hospital websites and 78% of the sites whose main focus was transcatheter procedures.

A sample of websites and their scores (five best, five worst and five in between) is given in Table 1.

**Table 1.** Scores achieved by a sample of websites assessed

URL	Score
http://www.nhsdirect.wales.nhs.uk/encyclopaedia/a/article/aorticvalvereplacement/	14
http://www.nhs.uk/conditions/Aorticvalvereplacement/Pages/Whatisitpage.aspx	13
http://www.nhsinform.co.uk/health-library/articles/a/aorticvalvereplacement/whatisitpage/	13
http://www.healthgrades.com/procedures/heart-valve-replacement	12
http://heart.uvahealth.com/services/cardiac-valve-center/treatment-and-research/aortic-valve-replacement	12
http://www.hopkinsmedicine.org/healthlibrary/test_procedures/cardiovascular/heart_valve_repair_or_replacement_surgery_92,P07975/	10
http://umm.edu/programs/heart/services/treatments/valve-disease-program/aortic-valve-repair	8
http://www.surgeryencyclopedia.com/A-Ce/Aortic-Valve-Replacement.html	5
http://www.bupa.co.uk/health-information/directory/h/heart-valve-disease	4
https://www.youtube.com/watch?v=ldvchT0LHF0	2
http://www.emoryhealthcare.org/medicaladvances/heart-vascular-etma/transcatheter-aortic-valve-implantation.html	0
http://www.universityhealthsystem.com/tavr-transcatheter-aortic-valve-replacement	0
http://cardiac.northshorelij.com/patient-services/tavr2	0
https://www.carilionclinic.org/heart-vascular/tavr-procedure	–1
http://www.sanfordhealth.org/services/transcatheteraorticvalvereplacement	–1

There were seven (9%) HON accredited sites and these had higher scores (median 7) than those without HON accreditation (median 4; *P*<0.05). There were also statistically significant differences in the scores between organisational/education and hospital websites (*P*<0.05), organisational/educational and commercial websites (*P*<0.05), and organisational/educational and video websites (*P*<0.05) are provided in Table 2. No website contained false information, but a number favoured a procedure or types of valve beyond established guidelines or had gaps in information typically about the postoperative recovery process. Categorisation of sites by TLD-types showed there were 52 .com/co.uk/.uk sites and these scored 5 (0 to 14), 18 .org sites which scored 4 (−1 to 11), four .gov sites which scored 8.5 (6 to 11) and five .edu sites which scored 4 (2 to 7). Statistical tests of these scores were underpowered due to small numbers.

**Table 2.** Comparison by category of web hosts

	Type of website				
	Organisational/educational, *n* = 33	Hospital, *n* = 24	Commercial, *n* = 8	Video, *n* = 14	Total, *n* = 79
Median score (range)	7 (2 to 14)	2 (−1 to 10)	2.5 (0 to 9)	5 (2 to 11)	5 (−1 to 14)
Health On the Net Foundation accredited, *n* (%)	4 (12)	2 (8)	0	1 (8)	7 (9)
Limitations, *n* (%)					
No information provided on: ● Recovery process ● Symptoms indicating surgery ● Intraoperative risks ● Postoperative complications ● Indications for type of prosthesis ● Evaluating benefits/disadvantages for each type of prosthesis	11 (33) 8 (24) 10 (30) 15 (45) 6 (18) 11 (33)	13 (54) 11 (46) 16 (67) 18 (75) 15 (63) 18 (75)	3 (38) 5 (63) 6 (75) 6 (75) 5 (63) 7 (88)	3 (23) 4 (31) 9 (69) 8 (62) 8 (62) 10 (77)	30 (38) 28 (36) 41 (53) 47 (60) 34 (44) 46 (59)
Bias in favour of: ● A procedure (Transcatheter aortic valve replacement / minimally invasive procedure) ● A type and/or make of prosthetic valve	10 (30) 1 (3)	11 (46) 1 (4)	1 (13) 2 (25)	2 (15) 0	24 (31) 4 (5)
Contains false information	0	0	0	0	0

## Discussion

### Summary

Web-based information on aortic valve replacement is highly variable with scores between −1 and 14. The educational sites had the highest median score (7) but scores ranged from 2 to 14. Hospital sites had scores (median 2) similar to the commercial sites (median 2.5).

### Strengths and limitations

The ideal study may have been to ask a number of patients, say 100, to search freely and for the research team to then score the sites they read. The methodology used was far simpler, and cannot have mimicked a potentially idiosyncratic individual search. The authors expect that they have accessed all sites that a patient would likely find by using the four most popular search engines and looking at the first 50 results from each.

### Comparison with the literature

The quality of websites is known to be variable^4–7^ although there is surprisingly little work on medical sites despite their obvious potential relevance to patients. A web-based study^9^ has shown that information about ‘murmur’ is often inaccurate tending to stress the worst possible outcome and rarely informing that a benign flow murmur is the most common cause.

### Implications for practice

Patient empowerment requires information for which the internet is a major resource. However, there are a number of problems faced by patients seeking information. The first is determining the reliability of a site. HON accreditation requires high governance standards and HON-accredited sites had higher median scores than the others. Only 7(9%) of sites were HON-accredited and many of the best sites were not HON-accredited. HON criteria focus on accessibility, accountability, author qualifications, and overall good governance and do not assess the reliability of specialist information provided. The authors suggest that HON should collaborate with the specialist professional societies relevant to the website being evaluated to add a further criterion concerned with clinical reliability. A patient may expect hospital sites to be reliable but these tended to be biased towards the specialist procedures offered by the hospital rather than offering more comprehensive information.

Another problem is finding a site. The best and most reliable did not appear on the top page, for example the best Yahoo site was ranked 63^rd^. During the preparation of this report, a new site was launched by BUPA and the Society for Cardiothoracic Surgery in Great Britain and Ireland (SCTS) (http://scts.org/patients/having-heart-surgery/). It scored 12 so was among the best sites but still does not appear on search engines.

Most sites contained limitations, most frequently about possible complications of surgery and the recovery process. The reasons for choosing one type of prosthesis over another were often not discussed including the possible limitations of warfarin and concerns around pregnancy. None of the sites discussed the psychological effects of surgery and there was little reference to support groups. Clinicians need to be aware of these gaps and provide additional complementary information at the same time as directing patients to the highest scoring sites which were the BUPA/SCTS site and the three NHS sites. The NHS sites contained similar information but on different domains: nhsdirect.wales.nhs.uk; nhs.uk; and nhsinform.co.uk. It would seem economic to combine these or at least develop cross-links to reduce maintenance costs.

## Appendix

**Appendix 1.** Scoring criteria

**Positive**
Multiple types of grafts: biological and mechanical with guide to who has which (age >65 years biological) 0 – Mentions only one graft 1 – Mentions all but does not offer guide 2 – Mentions all and offers guide Multiple types of procedure: Thoracotomy, minimally invasive procedure, transcatheter aortic valve replacement 0 – Mentions only one method 1 – Mentions some 2 – Mentions all Advantages and disadvantages of biological and mechanical (warfarin, clicking, deterioration) 0 – No evaluation given 1 – Partial evaluation given (for example, advantages of both or advantages and disadvantages of one) 2 – Full evaluation given In op complications 0 – Does not mention 1 – Mentions some and does not offer evaluation 2 – Comprehensive with evaluation Post op complications (heartblock, thromboembolism, deterioration) 0 – Does not mention 1 – Mentions some and does not offer evaluation 2 – Comprehensive with evaluation Indications for surgery —﻿ symptoms 0 – Does not mention 1 – Mentions some and does not offer evaluation 2 – Comprehensive with evaluation Recovery (time off work usually 2–3 months, valve noise) 0 – Does not mention 1 – Mentions some and does not offer evaluation 2 – Comprehensive with evaluation
**Negative**
Misleading in terms of main procedure (for example, transcatheter procedure, minithoracotomy as mainstay) 0 – Not evident 1 – Unclear 2 – Clearly supporting one form of procedure Bias towards type/make of graft 0 – Not evident 1 – Partially supports one or a few type(s)/make(s) of valve 2 – Clearly supporting one type/make of valve Provides false information of any sort 0 – Not evident 1 – Occurs once 2 – Occurs more than once Scoring system designed based on: Information known to be important and likely to be of interest to a patient Previously published test for reliability of internet information^12^ Aspects of HON guideline criteria^11^

**Appendix 2.** List of websites evaluated

Google http://www.nhs.uk/conditions/Aorticvalvereplacement/Pages/Whatisitpage.aspx Google https://en.wikipedia.org/wiki/Aortic_valve_replacement Google http://www.webmd.com/heart/aortic-valve-replacement-surgery Google https://www.youtube.com/watch?v=5jLfPlQBYuw Google https://www.nlm.nih.gov/medlineplus/ency/article/007407.htm Google http://my.clevelandclinic.org/services/heart/disorders/valvetreatment/aorticvalvesurgery Google http://www.texasheart.org/HIC/Topics/Proced/vsurg.cfm Google http://www.heart.org/HEARTORG/Conditions/More/HeartValveProblemsandDisease/Options-for-Heart-Valve-Replacement_UCM_450816_Article.jsp Google http://www.heartsurgeons.com/procedures3.html Google http://www.cts.usc.edu/aorticvalvereplacement.html Google http://www.nhsdirect.wales.nhs.uk/encyclopaedia/a/article/aorticvalvereplacement/ Google http://www.onxlti.com/message-to-patients/tissue-or-mechanical-heart-valve/ Google http://www.surgeryencyclopedia.com/A-Ce/Aortic-Valve-Replacement.html Google http://www.sts.org/patient-information/valve-repair/replacement-surgery/aortic-valve Google http://www.papworthhospital.nhs.uk/content.php?/our_services/cardiac_services_heart/avr Google http://www.heart-valve-surgery.com/aortic-valve-replacement-surgery.php Google http://www.mayoclinic.org/diseases-conditions/aortic-stenosis/basics/treatment/con-20026329 Google http://www.hopkinsmedicine.org/heart_vascular_institute/conditions_treatments/treatments/valve_aortic.html Google http://newheartvalve.com/treatment-options Google https://www.surgery.medsch.ucla.edu/cardiac/Clinical_Aortic%20Valve%20Replacement.shtml Google http://emedicine.medscape.com/article/903836-overview Google http://www.webmd.boots.com/heart-disease/guide/valve-disease-treatment Google http://www.yourheartvalve.com/productinformation/pages/aorticvalveporcine.aspx Google http://www.edwards.com/procedures/aorticstenosis/Pages/aorticstenosis.aspx Google http://umm.edu/programs/heart/services/treatments/valve-disease-program/aortic-valve-repair Google http://www.ctsnet.org/article/aortic-valve-replacement-edwards-intuity-sutureless-bioprosthesis-through-right-anterior Yahoo https://www.bhf.org.uk/heart-health/treatments/valve-heart-surgery.aspx Yahoo https://www.nlm.nih.gov/medlineplus/ency/article/002954.htm Yahoo http://www.bmihealthcare.co.uk/treatment/treatmentsdetail?p_name=Aortic%20valve%20replacement&p_treatment_id=185 Yahoo http://www.bupa.co.uk/health-information/directory/h/heart-valve-surgery Yahoo https://en.wikipedia.org/wiki/Aortic_valve Yahoo http://heart.emedtv.com/aortic-valve-replacement/aortic-valve-replacement.html Yahoo http://www.nhsinform.co.uk/health-library/articles/a/aorticvalvereplacement/whatisitpage/ Yahoo http://www.hopkinsmedicine.org/healthlibrary/test_procedures/cardiovascular/heart_valve_repair_or_replacement_surgery_92,P07975/ Yahoo https://www.bhf.org.uk/heart-matters-magazine/medical/valve-disease Yahoo http://www.healthgrades.com/procedures/heart-valve-replacement Yahoo http://www.heartfoundation.org.au/SiteCollectionDocuments/Heart-valve-surgery.pdf Yahoo http://www.medtronic.com/patients/heart-valve-disease/after-surgery/index.htm Yahoo https://www.floridahospital.com/aortic-valve-replacement Yahoo http://www.bupa.co.uk/health-information/directory/h/heart-valve-disease Bing http://www.webmd.com/heart-disease/video/aortic-valve-replacement Bing http://emedicine.medscape.com/article/780702-overview Bing http://www.rbht.nhs.uk/patients/why-us/patients-stories/keyhole-surgery/ Ask Jeeves https://www.nlm.nih.gov/medlineplus/ency/article/007408.htm Ask Jeeves http://www.harthosp.org/heart/tavr/default.aspx Ask Jeeves http://wexnermedical.osu.edu/patient-care/healthcare-services/heart-vascular/conditions-treatments/aortic-valve-replacement Ask Jeeves http://heart.uvahealth.com/services/cardiac-valve-center/treatment-and-research/aortic-valve-replacement Ask Jeeves http://www.emoryhealthcare.org/medicaladvances/heart-vascular-etma/transcatheter-aortic-valve-implantation.html Ask Jeeves http://www.upmc.com/services/heart-vascular/services/programs/Pages/center-aortic-valve-disease.aspx Ask Jeeves https://www.utswmedicine.org/conditions-specialties/heart/programs/cardiothoracic-surgery/transcatheter-aortic-valve-replacement.html Ask Jeeves http://www.stvincents.org/medical-services/cardiology/regional-heart-center-services/aortic-valve-replacement-tavr Ask Jeeves https://www.uahealth.com/services/heart-surgery/transcatheter-aortic-valve-replacement-tavr Ask Jeeves https://www.dukemedicine.org/treatments/heart/aortic-valve-disease Ask Jeeves https://www.scripps.org/services/heart-care__interventional-cardiology/transcatheter-aortic-valve-replacement-tavr Ask Jeeves https://www.floridahospital.com/tampa/cardiovascular/valve-center-program/transcatheter-aortic-valve-replacement-tavr Ask Jeeves http://heartcaremw.com/services/heart-valve-clinics/tavr.html Ask Jeeves http://heart.ucla.edu/body.cfm?id=170 Ask Jeeves https://www.sharecare.com/health/heart-surgeries/what-risks-aortic-valve-replacement Ask Jeeves http://www.universityhealthsystem.com/tavr-transcatheter-aortic-valve-replacement Ask Jeeves http://www.cigna.com/healthwellness/hw/medical-topics/aortic-valve-stenosis-uf4513abc Ask Jeeves https://www.carilionclinic.org/heart-vascular/tavr-procedure Ask Jeeves http://www.hoag.org/Specialty/Heart-and-Vascular-Institute/Pages/Transcatheter-Aortic-Valve-Replacement-TAVR/Transcatheter-Aortic-Valve-Replacement-TAVR.aspx Ask Jeeves http://cardiac.northshorelij.com/patient-services/tavr2 Ask Jeeves http://www.sanfordhealth.org/services/transcatheteraorticvalvereplacement Ask Jeeves http://www.medstarunionmemorial.org/our-services/heart-care/treatments/surgery/transcatheter-aortic-valve-replacement-tavr/#q={} Ask Jeeves http://www.cedars-sinai.edu/Patients/Programs-and-Services/Heart-Institute/Centers-and-Programs/Cardiothoracic-Surgery-Services/Heart-Valve-Repair-and-Replacement/ Ask Jeeves http://www.betterhealth.vic.gov.au/bhcv2/bhcmed.nsf/pages/CT02lite_en Youtube https://www.youtube.com/user/BritishHeartFound?v=fIbwK9_DKfU Youtube https://www.youtube.com/watch?v=C-sIdppyaPQ Youtube https://www.youtube.com/watch?v=m5DnlWun8oE Youtube https://www.youtube.com/watch?v=9m1zMWymn2U Youtube https://www.youtube.com/watch?v=Nae1hWRGpPQ Youtube https://www.youtube.com/watch?v=MmOadWd8Lys Youtube https://www.youtube.com/watch?v=w8FxMEgIxUk Youtube https://www.youtube.com/watch?v=2vf9TwktzTk Youtube https://www.youtube.com/watch?v=2gZX-MjcKnw Youtube https://www.youtube.com/watch?v=ldvchT0LHF0 Youtube https://www.youtube.com/watch?v=_icWNXzTLsk Youtube https://www.youtube.com/watch?v=psE7nGhaXOY
